# Chromosome segregation in plant meiosis

**DOI:** 10.3389/fpls.2014.00279

**Published:** 2014-06-17

**Authors:** Linda Zamariola, Choon Lin Tiang, Nico De Storme, Wojtek Pawlowski, Danny Geelen

**Affiliations:** ^1^Department of Plant Production, Faculty of Bioscience Engineering, University of GhentGhent, Belgium; ^2^Department of Plant Breeding and Genetics, Cornell University, Ithaca, NY, USA

**Keywords:** chromosome segregation, cohesion, kinetochore, meiosis, plant, recombination, spindle, synapsis

## Abstract

Faithful chromosome segregation in meiosis is essential for ploidy stability over sexual life cycles. In plants, defective chromosome segregation caused by gene mutations or other factors leads to the formation of unbalanced or unreduced gametes creating aneuploid or polyploid progeny, respectively. Accurate segregation requires the coordinated execution of conserved processes occurring throughout the two meiotic cell divisions. Synapsis and recombination ensure the establishment of chiasmata that hold homologous chromosomes together allowing their correct segregation in the first meiotic division, which is also tightly regulated by cell-cycle dependent release of cohesin and monopolar attachment of sister kinetochores to microtubules. In meiosis II, bi-orientation of sister kinetochores and proper spindle orientation correctly segregate chromosomes in four haploid cells. Checkpoint mechanisms acting at kinetochores control the accuracy of kinetochore-microtubule attachment, thus ensuring the completion of segregation. Here we review the current knowledge on the processes taking place during chromosome segregation in plant meiosis, focusing on the characterization of the molecular factors involved.

## Introduction

Meiosis is a specialized cell division that generates four haploid daughter cells from a diploid parent cell after a single round of DNA replication and two consecutive rounds of nuclear division. In the first nuclear division, homologous chromosomes segregate (reductional cell division), and in the second one, sister chromatids segregate (equational cell division). As such, each daughter cell carries half the amount of the parental genetic material. The accurate segregation of chromosomes during meiosis is essential for the formation of haploid gametes. Failure in the proper execution of chromosome segregation inevitably leads to the formation of imbalanced gametes and aneuploid or polyploid progeny. In plants, aneuploidy is more tolerated than in animals and viable aneuploid plants have been observed, especially among the progeny of triploid individuals (Henry et al., 2005, 2010). Despite being affected in growth and reproduction (Birchler et al., 2001), aneuploids may have an evolutionary role, serving as a bridge to euploid polyploid plant formation through repeated generations of selfing (Ramsey and Schemske, 1998; Henry et al., 2005). Polyploid plants generated through aneuploids or by the polyploidization events of somatic doubling and unreduced gametes, are considered as a prominent driving force in plant genome evolution (Ramsey and Schemske, 2002; Adams and Wendel, 2005; Comai, 2005; Otto, 2007).

To ensure the correct completion of the meiotic cell division program, a sequence of coordinated steps must take place during the two phases of meiosis. In meiosis I, homologous chromosomes must pair and synapse and physically exchange genetic material through recombination. The resulting points of crossing-over, also termed chiasmata, form links between the two homologs in the bivalent configuration and ensure proper positioning of the bivalent relative to the division spindle and balanced segregation of homologs in anaphase I. Additionally, to achieve this, sister kinetochores from each homolog must attach to microtubules emanating from the same spindle pole, a process called monopolar kinetochore attachment, and cohesion must be lost in a stepwise manner. More specifically, at anaphase I, cohesion is released at chromosome arms but not at sister centromeres, allowing homologs to segregate without affecting the physical connection between both sister chromatids. In meiosis II, chromosome segregation in the two resulting haploid interphase nuclei occurs in an equational manner and hence strongly resembles the dynamics of a mitotic cell division. Cohesion at centromeres is retained until anaphase II to ensure bipolar attachment of sister kinetochores to microtubules and equational segregation of chromatids into four haploid daughter cells. Progression through the meiotic cell division is regulated at determined checkpoints by the activity of CDK (Cyclin-Dependent Kinase) - cyclin complexes and the Anaphase Promoting Complex/Cyclosome (APC/C) (Harper et al., 2002; Cooper and Strich, 2011). In particular, the Spindle Assembly Checkpoint (SAC) acts during the transition between metaphase and anaphase of the two meiotic cell divisions to ensure correct kinetochore-microtubule attachments and faithful chromosome segregation (Malmanche et al., 2006; Yamamoto et al., 2008). In plants, checkpoints appear to be less stringent compared to yeast and animals, since completion of meiosis is achieved in several meiotic mutants creating imbalanced gametes (Wijnker and Schnittger, 2013).

Most of the knowledge on the molecular biology of mitotic and meiotic chromosome segregation comes from studies in yeast (reviewed in Marston, 2014). However, the mechanisms of chromosome segregation are conserved in eukaryotes, including plants (Dawe, 1998; Bhatt et al., 2001). In the last decade, the increasing availability of genomic tools and the development of *Arabidopsis thaliana*, but also maize (*Zea mays*) and rice (*Oryza sativa*), as model systems, have led to the identification of a large number of conserved meiotic genes (Mercier and Grelon, 2008). Phenotypic and cytogenetic analyses of the corresponding mutants, have unraveled the function of several molecular factors required for proper chromosome segregation in plants (Bhatt et al., 2001; Ma, 2006). Therefore, the focus of this review will be on the major cellular processes that take place to ensure accurate chromosome segregation in plant meiosis and the related genes that have been yet identified in *Arabidopsis*, maize and rice. Other factors having an effect on chromosome segregation in plant meiosis, such as environmental stresses and changes in ploidy level have been described in recent reviews (Comai, 2005; Madlung and Wendel, 2013; De Storme and Geelen, 2014). After mentioning the importance of homologous chromosome pairing and recombination, two subjects extensively discussed in other reviews (Hamant et al., 2006; Edlinger and Schlögelhofer, 2011; Osman et al., 2011; Tiang et al., 2012; Da Ines et al., 2014), we describe the relevance of cohesion, focusing on the roles of the cohesin complex and on the cohesion dynamics (e.g., loading, release and protection) during meiotic cell division. Next, we discuss the role of centromeric and kinetochore proteins in establishing proper spindle attachment during meiosis I and II, and additionally describe what is currently known on the checkpoint control mechanisms acting at kinetochores. Finally, we report the molecular mechanisms underlying microtubule organization and we focus on the relevance of spindle orientation in plant meiosis.

## Homologous pairing and recombination as a basis for reductional cell division in meiosis i

### Homologous chromosome pairing and synapsis

To ensure accurate segregation, chromosomes must first recognize their homologous partners and pair with them during early meiotic prophase I. This process leads to the formation of bivalents, which ensures correct bipolar attachment of homologous centromeres to the division spindle at metaphase I in a way that each of the chromosomes in the bivalent moves to a different pole at anaphase I. Bivalent formation is also required for proper positioning of chromosomes at the metaphase plate. Consequently, mutants with chromosome pairing problems exhibit chromosome segregation defects (Bozza and Pawlowski, 2008).

It is assumed that chromosome homology recognition is based on their DNA sequence. Although mechanisms that bring homologous chromosomes together have yet to be fully elucidated, studies in a variety of species, including plants, have shown that chromosome pairing is strongly dependent on their dynamics in early meiotic prophase as well as the initiation and progression through the early stages of the recombination pathway. Chromosome dynamics in prophase I is largely controlled by the behavior of telomeres, blocks of highly conserved repetitive DNA sequence at the ends of chromosomes (Siderakis and Tarsounas, 2007). Telomeres attach to the nuclear envelope before the onset of chromosome pairing, and gather on a small region, forming a unique structure that resembles a flower bouquet, the so called telomere bouquet (Bass et al., 2000; Golubovskaya et al., 2002; Harper et al., 2004; Richards et al., 2012). The bouquet arrangement has been observed in most eukaryotes (Klutstein and Cooper, 2014). The exact role of the bouquet is still being debated. However, mutants defective in bouquet formation are frequently also defective in chromosome pairing, which implies a role of the bouquet in this process (Harper et al., 2004; Klutstein and Cooper, 2014). One example of such mutant is *plural abnormality of meiosis 1* (*pam1*) in maize, which exhibits significant reduction in homologous pairing (Golubovskaya et al., 2002). In this mutant, telomeres attach to the nuclear envelope but fail to cluster. The bouquet formation has been, therefore, suggested to promote homologous paring by bringing chromosome ends together (Harper et al., 2004).

Alternative chromosome interaction mechanisms have been described in several species, including *Caenorhabditis elegans* and *Arabidopsis* (Armstrong et al., 2001; Phillips and Dernburg, 2006). In *C. elegans*, telomeres do not form the bouquet but pairing centers, short chromosome segments recognized by specific zinc-finger proteins, that attach to the nuclear envelope during early prophase I, also bringing homologous chromosomes together (Phillips and Dernburg, 2006). In *Arabidopsis*, telomeres cluster in meiotic interphase on the nucleolus rather than the nuclear envelope (Armstrong et al., 2001). Subtelomeric regions of *Arabidopsis* chromosomes start to pair before telomeres dissociate from the nucleolus, suggesting that the clustering on the nucleolus may play a role similar to that of the canonical bouquet. *Arabidopsis* telomeres establish their connections with the nuclear envelope during leptotene and zygotene, although without an obvious bouquet formation (Armstrong et al., 2001).

Interestingly, the connections used to attach chromosomes to the nuclear envelope in *C. elegans* and *Arabidopsis* are homologs of the same transmembrane proteins that are used in other species to tether telomeres to the nuclear envelope during bouquet formation. SUN domain proteins, identified in yeast, mammals, *C. elegans*, maize, as well as *Arabidopsis*, cross the inner nuclear membrane (Chikashige et al., 2007; Schmitt et al., 2007; Penkner et al., 2009; Sato et al., 2009; Graumann et al., 2010; Murphy et al., 2010). They interact at their N-termini with telomere binding proteins while their C-termini bind transmembrane proteins containing a conserved KASH domain that cross the outer membrane and interact with the cytoskeleton (Miki et al., 2004; Zhou et al., 2012). The commonality of the structures attaching telomeres to the nuclear envelope reinforces the notion that the telomere-nuclear membrane attachments in *C. elegans* and *Arabidopsis* may be functionally similar to the presence of the canonical bouquet.

It has been shown in several species that the cytoskeleton acts through the telomere-nuclear membrane attachments to induce dynamic motility of chromosomes (Bhalla and Dernburg, 2008; Koszul et al., 2009; Sheehan and Pawlowski, 2009; Woglar and Jantsch, 2013). The chromosome movements are thought to help the chromosomes to engage in finding their pairing partners as well as resolving their entanglements.

Another process, which is required for proper chromosome segregation, and closely follows chromosome pairing, is synapsis. Synapsis is installation of a proteinaceous structure, the synaptonemal complex (SC), between the paired homologous chromosomes, which stabilizes the pairing interactions. The SC consists of two lateral elements (LEs) which reside at the base of the chromosome loops and are held together in parallel by transverse filament proteins. In most eukaryotes, the LEs are derived from the axial elements (AEs) loaded on the chromosomal axis before synapsis. Installation of the synaptonemal complex is also closely linked with the formation of crossovers (see the following section), and so synapsis also affects chromosome segregation through its role in crossover formation. *Arabidopsis* mutants defective in synaptonemal complex formation exhibit univalents at metaphase I and improper chromosome segregation at anaphase I (Ross et al., 1997; Higgins et al., 2005).

### Meiotic recombination

Meiotic recombination affects segregation of chromosomes in at least two ways. First, studies in many species, including plants, mammals, and fungi, have indicated that homologous chromosome pairing is closely connected to meiotic recombination (Pawlowski and Cande, 2005). Second, crossovers, reciprocal chromosome segment exchanges formed as a result of meiotic recombination, form physical connections, known as chiasmata, between homologous chromosomes in each bivalents. Chiasmata keep bivalents together to ensure proper orientation and segregation of chromosomes during the first meiotic division.

Recombination in meiosis is initiated by the formation of double strand breaks (DSBs) in chromosomal DNA, triggered by Spo11, a conserved topoisomerase type-II-like protein (Keeney et al., 1997). The MRN complex (MRE11/RAD50/NBS1) then resects the breaks creating single-stranded DNA overhangs (Borde, 2007), which then invade appropriate regions on the homologous chromosomes. This process is promoted by two recombination proteins, Rad51 and Dmc1 (Masson and West, 2001). Rad51 is solely responsible for the repair of DNA breaks using sister chromatids as templates. However, this process is restrained and replaced by repair via the homologous chromosome when Dmc1 is localized to meiotic DNA break sites together with Rad51 (Bishop et al., 1992; Niu et al., 2009). In *Arabidopsis*, mutating *Rad51* results in chromosome fragmentation (Li et al., 2004). However, fragmentation is not observed in the *dmc1* mutant (Couteau et al., 1999). These observations suggest that the function of Dmc1 is distinct from Rad51, as Dmc1 promotes interhomolog recombination rather than intersister recombination (Kurzbauer et al., 2012; Pradillo et al., 2012).

Meiotic recombination results in formation of crossovers and non-crossovers (which include gene conversions). The number and location of crossovers are tightly regulated. In most plant species, only one to four crossovers are formed per bivalent (Crismani and Mercier, 2012). At least one crossover must be formed per bivalent to ensure correct chromosome segregation at anaphase I. However, the number of crossovers per chromosome is limited by crossover interference, a mechanism that prevents formation of crossovers next to each other (Jones, 1984). A group of proteins called ZMM, which contains Zip1, Zip2, Zip3, Zip4, Msh4, Msh5, and Mer3, have been identified as essential for the formation of interference-dependent crossovers in yeast (Börner et al., 2004). Homologs of several of these proteins have been studied in *Arabidopsis* and found to play similar roles in crossover formation (Higgins et al., 2004, 2005, 2008; Chen et al., 2005; Mercier et al., 2005; Chelysheva et al., 2007). Loss of MSH4 in *Arabidopsis*, results in a reduction in crossover frequency to 15% of the wild-type level (Higgins et al., 2004). Similar effect was shown in the *Arabidopsis mer3* mutant (Chen et al., 2005; Mercier et al., 2005). Interestingly, the ZMM group includes proteins that are primary components of the synaptonemal complex, such as ZIP1. This interdependence indicates a link between crossover formation and synapsis. Overall, about 85% of *Arabidopsis* crossovers arise from the interference-dependent pathway (Higgins et al., 2004). The remaining crossovers are interference-independent, and are generated by a distinct group of proteins including MUS81 and EME1/MMS4 (Berchowitz et al., 2007).

Recombination events, including crossovers are not distributed randomly along chromosomes. Instead they tend to appear at certain chromosomal locations known as recombination hotspots (Drouaud et al., 2006). In plant species with large genomes, such as maize, barley, or wheat, crossovers are predominantly present in chromosome regions close to the telomeres (Akhunov et al., 2003; Gore et al., 2009). Crossover distribution affects the positions of chiasmata and may have implications for bivalent stability and chromosome segregation. However, neither mechanisms that control crossover distribution nor implications of crossover distribution for chromosome behavior in meiosis are well understood.

### Early defects in chromatin structure have an impact on homologous chromosome segregation: ASK1

ASK1 (*Arabidopsis* SKP1-like1) encodes one of the 21 predicted *Arabidopsis* homologs of the yeast and human Skp1 proteins (Yang et al., 1999; Zhao et al., 2003a,b). Skp proteins are an essential component of the Skp1-Cullin-F-box (SCF) complex, that belongs to a class of E3 ubiquitin ligases that target a variety of proteins for ubiquitin-mediated degradation via the 26S proteasome pathway (Petroski and Deshaies, 2005). ASK1 is the Skp homolog that has been best characterized in *Arabidopsis*. *ask1-1* mutants display defects in plant growth, flower development and male fertility (Yang et al., 1999; Zhao et al., 2001, 2003b). Male sterility arises from meiotic defects in prophase I that lead to erroneous homologous chromosome segregation in meiosis I and sister chromatid segregation in meiosis II, and to the subsequent formation of unbalanced spores. During prophase I, chromosomes maintain a leptotene-like structure with long and thin threads that do not synapse, as demonstrated by the absence of the typical SC structure (Wang et al., 2004). FISH experiments using a centromeric probe showed the presence of more than 5 signals in *ask1-1* meiocytes during pachytene, confirming lack of homologous pairing and bivalents formation (Zhao et al., 2006). The localization of the α-kleisin subunit of the cohesin complex SYN1 (described in the next paragraph) was also found to be altered in *ask1* meiocytes from zygotene to anaphase I. These observations together with a premature sister chromatid detachment detected by FISH in anaphase I, suggest that *ask1* mutation alters cohesin distribution and function, which is necessary for proper pairing and synapsis (Zhao et al., 2006). The abnormalities detected in *ask1* seem to derive from early defects in meiotic chromatin structure and chromosome reorganization in leptotene that cause a prolonged attachment of chromosomes to the nuclear membrane and the nucleolus, alterations in rDNA structure, prolonged attachment of the telomeres to the nucleolus, and defects in histone 3 acetylation, overall leading to the absence of homologous chromosome pairing (Yang et al., 2006). Hence, ASK1 is most likely required for chromosome conformation and remodeling of meiotic chromosomes by controlling the release of chromatin from the nucleolus and nuclear membrane starting from leptotene (Yang et al., 2006). Several hypotheses have been currently proposed to explain the potential role of ASK1 in meiosis, consistent with the meiotic defects observed in the mutant and the homology of ASK1 to Skp proteins (Yang et al., 2006; Zhao et al., 2006). ASK1 may control the degradation of a protein which inhibits the leptotene to zygotene transition, so that the alterations observed in chromatin structure and organization would be a consequence of the block of this transition. Alternatively, ASK1 might regulate the interaction of chromosomes to the nuclear membrane by degrading one or more proteins that link chromatin to the nuclear matrix, thus allowing a nuclear reorganization during leptotene and zygotene. ASK1 may also control chromatin structure by regulating chromatin remodeling proteins, as suggested by the alterations detected in histone 3 acetylation. However, the specific function of ASK1 in male meiosis is not yet defined.

## Sister chromatid cohesion is essential for faithful chromosome segregation

### The cohesin complex

**Figure 1:**
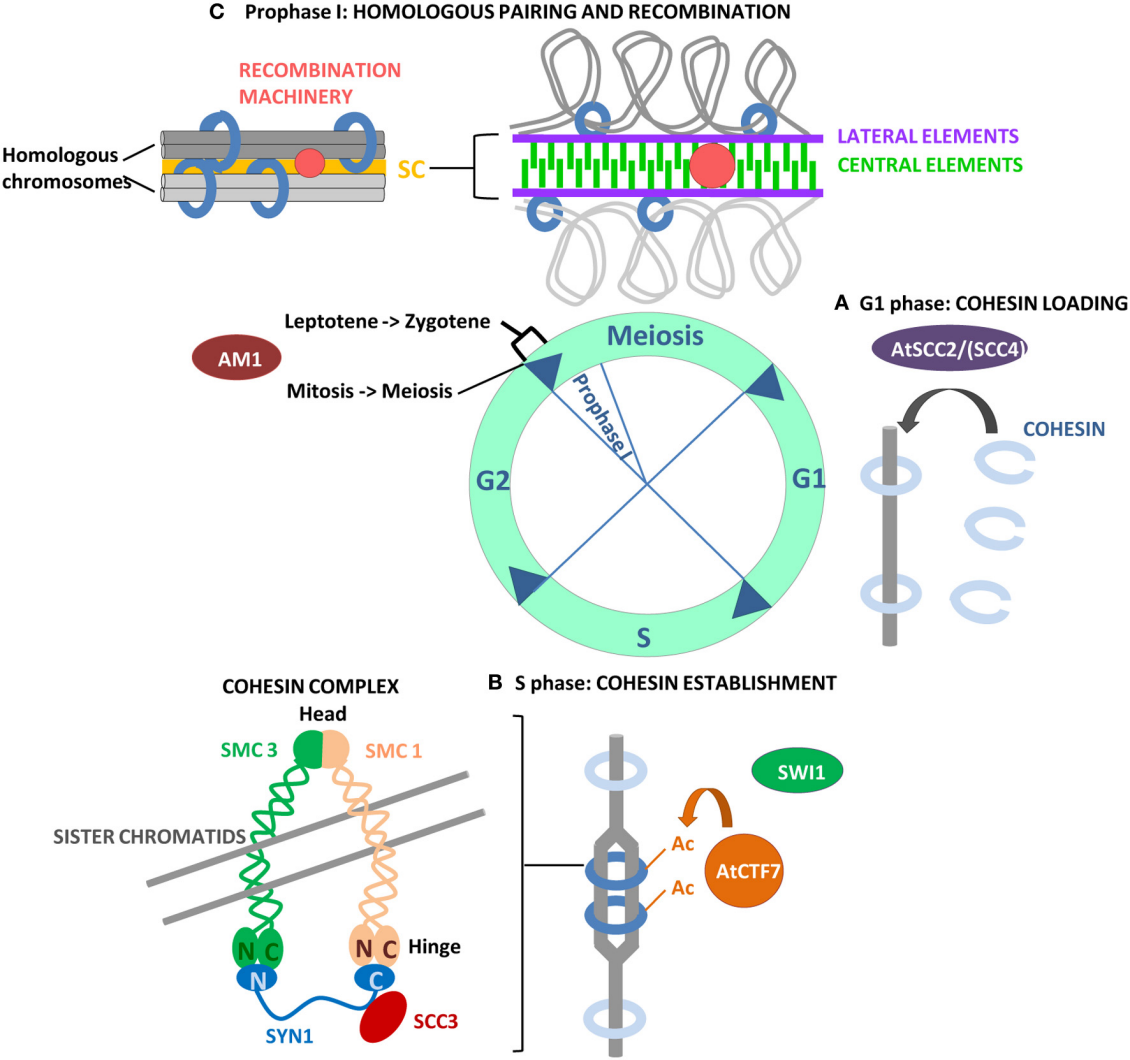
**Overview of the events that allow the establishment of the cohesin complex on chromosomes, necessary for the successive steps of chromosome segregation, including homologous pairing and recombination in meiosis I. (A)** Loading of cohesin on chromosomes requires the SCC2/SCC4 complex, only AtSCC2 has been characterized in *Arabidopsis*. **(B)** The establishment of chromosome cohesion takes place during DNA replication in S phase when Eco1/CTF7 acetylates SMC3 residues, effectively closing the cohesin ring. AtCTF7 has acetylatransferase activity *in vitro* and is required for the establishment of chromosome cohesion in *Arabidopsis*. SWI1 is an *Arabidopsis* protein with a role in cohesin establishment even if the mechanism of action is not yet known. Its maize homolog AM1 is required for the switch from mitosis to meiosis and for a putative checkpoint between leptotene and zygotene in prophase I. **(C)** Cohesion is required for SC elongation and polymerization and for meiotic recombination in prophase I.

Sister chromatids must be held together from the moment of their synthesis in S-phase until their separation in anaphase to ensure correct attachment of chromosomes to the spindle and accurate chromosome segregation in dividing cells. Cohesin is the multi-subunit protein complex that mediates sister chromatid cohesion in meiosis and mitosis by physically trapping them in a tripartite ring structure (Haering et al., 2008). The complex is highly conserved in eukaryotes and is composed of a core of four evolutionary conserved proteins, extensively studied in yeast and animals. In mitosis, the cohesin complex is composed of two members of the SMC family (structural maintenance of chromosomes), SMC1 and SMC3, and two auxiliary SCC subunits (sister chromatid cohesion), the α-kleisin RAD21/SCC1 and SCC3. In meiosis, the structure of the cohesin complex is highly similar, except for the RAD21/SSC1 component, which is replaced by its counterpart Rec8 (Stoop-Myer and Amon, 1999; Watanabe and Nurse, 1999). SMC1 and SMC3 consist, in their folded configuration, of a globular head and a hinge domain, connected by a long anti-parallel coiled coil. The proposed model of action of cohesin, the embrace model, requires the connection of the SMC hinge domains to form a SMC1/SMC3 heterodimer with a V-shaped structure, that can bind across sister chromatids and close, forming a ring, through a physical connection of the α-kleisin subunit to the C-terminal domain of SMC1 and the N-terminal domain of SMC3 (Gruber et al., 2003). The complex is stabilized by recruitment of SCC3 by the α-kleisin subunit (Figure 1) (for reviews on cohesin complex: Nasmyth and Haering, 2005; Onn et al., 2008; Peters et al., 2008).

#### SMC proteins

Similar as in yeast and animals, the sister chromatid connection in plants is also established through the cohesin complex. Homologs of the cohesin complex have been identified in some plant species and major progress on the understanding of their function has been achieved in the model plant Arabidopsis thaliana, in which all the components have been described. The Arabidopsis genome contains single copies of SMC1 and SMC3 cohesin subunits. Genetic studies revealed that loss of AtSMC1 or AtSMC3 functionality causes seedling lethality, hence impairing functional characterization (Liu et al., 2002). Localization studies using a specific antibody revealed that AtSMC3 is present in the cytoplasm and nucleus, on chromosomes and in the nuclear matrix of meiotic and mitotic cells, indicating a function in both types of cell divisions (Lam et al., 2005). At meiotic prophase, AtSMC3 localizes along sister chromatids to axial elements and lateral elements, similar to the Arabidopsis α-kleisin subunit SYN1. This observation confirms the conserved role of the cohesin complex in sister chromatid cohesion but also supports an additional function in SC formation, as proposed in yeast and mammals (Klein et al., 1999; Eijpe et al., 2000). By metaphase I, AtSMC3 localizes only to chromosome centromeres and, in addition, co-localizes to the spindle at metaphase I and anaphase I and II. The spindle localization is independent of SYN1 functionality and suggests that AtSMC3 might play an additional role as spindle associated protein, distinct from its conserved role in sister chromatid cohesion. This novel localization pattern is also conserved in mitosis and could be related to a role of AtSMC3 in spindle assembly and/or in the chromosome association with the spindle (Lam et al., 2005). A similar novel function has been suggested in human mitosis for the entire cohesin complex (Gregson et al., 2001).

Immunolocalization studies in tomato meiocytes (Solanum lycopersicum) revealed that SMC1 and SMC3 show a similar localization pattern as AtSMC3. In prophase I, SMC1 and SMC3 antibodies display a signal along AEs of the SC from leptotene to diplotene and a weak and diffuse signal on chromosomes at metaphase I and telophase II (Lhuissier et al., 2007). However, no localization to the spindle was documented, suggesting that the novel spindle function might be specific for Arabidopsis AtSMC3 and not conserved in other plant species.

#### Rec8 and SCC3

More intensive studies have been undertaken on the role of the meiotic α-kleisin subunit Rec8 in Arabidopsis (named SYN1 but also DIF1 and AtRec8), maize (AFD1), and rice (OsRad21-4/OsRec8) meiosis. In Arabidopsis, the homolog of Rec8, SYN1, is required for sister chromatid cohesion in meiosis (Cai et al., 2003; Chelysheva et al., 2005). SYN1 fully co-localizes with AtSCC3 at pachytene and is necessary for its proper loading on sister chromatids, confirming that they are indeed part of a complex (Chelysheva et al., 2005). FISH (fluorescence *in situ* hybridization) analysis on syn1 meiocytes using chromosome arm and centromeric probes show defective sister chromatid arm and centromere cohesion in meiosis I, confirming that SYN1 functions in cohesion (Cai et al., 2003).

In addition, SYN1 is required for synapsis of homologous chromosomes, being necessary for SC polymerization and elongation (Chelysheva et al., 2005). In syn1 meiocytes, synapsis is blocked and chromosome condensation and pairing are almost completely absent, leading to the presence of univalents at metaphase I (Bai et al., 1999; Bhatt et al., 1999). Localization of ASY1, a protein required for chromosome synapsis, recombination and SC assembly and widely used as a marker for chromosome axes in meiosis (Sanchez-Moran et al., 2008), is impaired in syn1 mutants, confirming the requirement of SYN1 for AE polymerization and elongation but not for their formation (Chelysheva et al., 2005). Synapsis is known to be closely related to meiotic recombination. Therefore, it is not surprising that SYN1 plays also a role in recombination, specifically in DSBs repair. Indeed, chromatin bridges and chromosome fragmentation are observed in syn1 meiosis I. They are suppressed by introducing into the syn1 mutant background the Atspo11 mutation, which abolishes DSBs formation and prevents recombination, confirming that SYN1 is required for DSBs repair (Chelysheva et al., 2005). Involvement of the cohesin complex in homologous chromosome pairing, assembly of the SC, and in meiotic recombination has been shown previously in other organisms. In yeast, Rec8 and SMC3 are required for SC formation and for repairing DSBs (Klein et al., 1999). In mouse, loss of Rec8 affects homologous recombination but does not affect SC formation and assembly. However, synapsis occurs between sister chromatids instead of homologous chromosomes, suggesting that Rec8 might define the chromosome unit and limit the SC binding sites to one single chromosome surface of a sister-chromatid pair in mammals (Xu et al., 2005).

Support for an additional role of Rec8 in homologous pairing and recombination in plants comes from studies on the maize α-kleisin subunit AFD1 and the rice OsRad21-4/OsRec8. A study on different afd1 alleles has revealed that AFD1 is required for AE installation, affecting the deposition of the recombination machinery on chromosomes (Golubovskaya et al., 2006). The rice OsRec8 regulates AE formation and may have a role in DNA DSBs repair, since localization of PAIR2 (homolog of Arabidopsis ASY1), ZEP1 (ZYP1 homolog), and MER3, involved in the formation of crossovers, is affected in Osrec8 mutants. As a consequence, no proper homologous pairing occurs (Zhang et al., 2006; Shao et al., 2011). Moreover, defective telomere bouquet formation is observed in Osrec8 and afd1 mutants, also preventing proper pairing of homologous chromosomes. Hence, OsRec8 regulates AE formation, homologous recombination and synapsis by affecting downstream proteins PAIR2, ZEP1, and MER3 (Shao et al., 2011).

Rec8 has a crucial role in the determination of kinetochore geometry for monopolar orientation in fission yeast, since rec8 mutants display loss of monopolar orientation at meiosis I and chromosome segregation defects (Yokobayashi et al., 2003; Sakuno et al., 2009). Similarly, Arabidopsis SYN1 is necessary for the monopolar attachment of sister kinetochores in meiosis I, as indicated by the observation of bipolar sister kinetochore attachment in meiosis I in the double syn1 Atspo11 mutant, in which syn1 chromosome fragmentation is suppressed allowing a clearer observation of chromosome segregation. However, the same defect in kinetochore orientation is observed for the other SCC cohesin subunit, AtSCC3, indicating that SYN1 is not sufficient for monopolar kinetochore orientation or, most likely, is inactive when the other members of the complex are not present. These data suggests that Rec8-containing cohesin complex is responsible for defining kinetochore geometry in meiosis I in plants, as proposed in yeast, Drosophila and mammals (Chelysheva et al., 2005; Watanabe, 2012).

AtSCC3 is the sole SCC3 homolog investigated in plants so far. It is required for normal plant growth and fertility and has a conserved role in proper sister chromatid cohesion, confirmed by the combination of univalents and bivalents observed in Atscc3 mutants (Chelysheva et al., 2005). However, in contrast to SYN1, AtSCC3 is not required for AE formation, since ASY1 localization in Atscc3 is normal and synapsis does not show major defects in the mutant. Moreover, only a low level of fragmentation is observed in Atscc3 and recombination is not defective, suggesting that the two SCC subunits, although being part of the same complex, may fulfill different additional functions (Chelysheva et al., 2005).

While AtSCC3 has no paralogs in the Arabidopsis genome, three α-kleisin homologs, SYN2, SYN3, and SYN4, are present that share about 38 % sequence similarity at their N-termini and 20 % at their C-termini with SYN1, and could partially compensate for each other (Schubert et al., 2009a). Two observations raise the hypothesis that the α-kleisin paralogs may be involved in cohesion in meiosis. First, in the syn1 Atspo11 double mutant, sister chromatid cohesion is only lost at anaphase I, suggesting that other homologs of the SYN1 family might be responsible for cohesion before that stage (Chelysheva et al., 2005). Second, SYN1 localization is only observed along chromosome axes but not at the core centromeres at metaphase I and metaphase II (Chelysheva et al., 2005; Cromer et al., 2013; Zamariola et al., 2013). It is known that SYN1, SYN2 and SYN4 may partially compensate for each other whereas SYN3 is required for plant viability, it localizes to the nucleolus and might have evolved a role in rDNA transcription and/or processing. A specific function in DNA repair in somatic cells has been suggested for SYN2, while SYN4 is required for centromere cohesion in mitosis (Schubert et al., 2009b). However, the role of the different paralogs is, at this time, not clear, and the creation of double or triple mutants might help unravelling their specific functions (Schubert et al., 2009b).

### Loading and establishment of chromosome cohesion

The loading of the cohesin complex onto chromosomes starts at telophase in humans and at the end of G1 in yeast and requires the evolutionary conserved SCC2/SCC4 complex (for reviews see Uhlmann, 2009; Ocampo-Hafalla and Uhlmann, 2011). Cohesin loading has been shown to be enriched at centromeric and pericentromeric regions promoting high fidelity chromosome segregation (Eckert et al., 2007). Recent studies in budding yeast have revealed that the observed enrichment is defined by the presence of the kinetochore subcomplex Ctf19, that promotes SCC2/SCC4 centromere association (Fernius et al., 2013). Also in Angiosperms, interphase nuclei show a preferred alignment of sister chromatids at centromeres, which might facilitate kinetochore bipolar orientation in mitosis, essential for correct chromosome segregation (Schubert et al., 2007). In plants, only the *Arabidopsis* homolog of the adherin SCC2 has been described. AtSCC2 is essential for plant viability and *Atscc2* plants show defects in embryogenesis and endosperm development (Schubert et al., 2009b; Sebastian et al., 2009). Using an inducible RNAi (RNA interference) system, Sebastian et al. (2009) demonstrated that AtSCC2 is required for sister chromatid cohesion and loading of the cohesin complex in meiosis, as indicated by defects in AtSCC3 localization. Furthermore, *Atscc2* mutants show an irregular localization of ASY1 and chromosome fragmentation, indicating that AtSCC2 is required for axial development and most likely for repair of DNA DSBs, supporting the notion that sister chromatid cohesion is a prerequisite for axial development and DSBs resolution (Sebastian et al., 2009).

The loading of cohesin is the first step through the establishment of sister chromatid cohesion that takes place during DNA replication. After the loading, cohesin is unstable due to the activity of the Wapl-Pds5 complex that promotes cohesin dissociation (Rowland et al., 2009). In yeast, cohesion is established during S-phase by the Eco1/CTF7 protein, that acetylates the SMC3 residues, effectively closing the cohesin ring (Rowland et al., 2009). In *Arabidopsis*, AtCTF7 exhibits acetyltranferase activity *in vitro* like its yeast and human homologs (Jiang et al., 2010). *Atctf7* homozygous mutants display a dwarf phenotype and aberrant microsporogenesis due to defects in chromosome segregation in mitosis and PMCs (pollen mother cells). FISH performed with a centromeric and a chromosome 4 arm probes on male meiocytes of *Atctf7* and AtCTF7 RNAi plants, revealed that the protein is required for both centromere and arm cohesion in meiosis (Bolaños-Villegas et al., 2013; Singh et al., 2013). Furthermore, localization of the cohesin complex subunits AtSMC3, AtSYN1 and AtSCC3 is impaired in *Atctf7* male meiosis, indicating that AtCTF7 is necessary for association of cohesin on chromatin in meiosis (Bolaños-Villegas et al., 2013; Singh et al., 2013). In addition, the level of expression of genes required for DNA repair is significantly altered in *Atctf7* mitotic and meiotic tissues, and the mutant plants show a lower ability to repair DNA double strand breaks *in vivo* in mitotic cells (Bolaños-Villegas et al., 2013). Taken together, these observations suggest that AtCTF7 is also required for DNA repair in *Arabidopsis*, as shown for Eco1 in yeast mitosis (Lu et al., 2010).

SWITCH1/DYAD (SWI1/DYAD) is an *Arabidopsis* protein with an essential role in the establishment of sister chromatid cohesion during early meiosis (Mercier et al., 2001, 2003). Different allelic mutations have been investigated for the *SWI1/DYAD* gene, all of them showing an impact on fertility due to different mechanisms affecting megasporogenesis (*swi1-1* and *dyad*; Motamayor et al., 2000; Siddiqi et al., 2000; Mercier et al., 2001; Agashe et al., 2002) or both mega and microsporogenesis (*swi1-2* and *dsy10*; Mercier et al., 2003; Boateng et al., 2008). *Swi1-1* and *swi1-2* alleles have been shown to have an effect on the female mitosis-meiosis switch, so that meiosis is converted into a mitotic cell division (Motamayor et al., 2000; Mercier et al., 2001). However, analysis of the *dyad* allele by Agashe et al. (2002) and Siddiqi et al. (2000) with a meiotic marker, provided evidence that the female megaspore enters the meiotic programme but does not progress into further meiotic divisions. Detailed studies of male meiosis for *swi1-2* and *dsy10* alleles, have shown that the mutants loose cohesion in a stepwise manner already in meiosis I, leading to the presence of 20 chromatids at metaphase I which segregate randomly in meiosis II, forming polyads (Mercier et al., 2001). Furthermore, the mutant lacks AE formation, leading to incorrect pairing and synapsis, and does not initiate recombination. These defects probably all derive from defective establishment of cohesion before the initiation of meiosis, since the protein is expressed exclusively in meiotic G1 and S phase (Mercier et al., 2003). Specifically, the localization of SYN1 in *swi1-2* meiocytes, indicates that SWI1 performs its function after the loading of the cohesin complex (Mercier et al., 2003). However, its specific function in chromosome cohesion is not yet understood.

Maize AM1 and rice OsAM1 are proteins closely related to SWI1. Mutants in *AM1* and *OsAM1* genes show defective sister chromatid cohesion, absence of homologous pairing and synapsis, and lack of homologous recombination (Pawlowski et al., 2009; Che et al., 2011). However, while *Arabidopsis swi1* mutants affect meiotic processes downstream of meiotic initiation and do not affect entrance in meiosis, maize *am1* mutants show typical features of mitotic division in the early steps of meiosis, indicating that AM1 is required for the transition from the mitotic cell cycle into meiosis. Meiocytes of a specific *am1* allele arrest during early meiotic prophase at the transition between leptotene and zygotene, suggesting the presence of a novel checkpoint in maize required for progression through prezygotene (Pawlowski et al., 2009). Similarly, in rice, OsAM1 is also likely involved in a checkpoint mechanism that regulates the transition from leptotene to zygotene (Che et al., 2011).

A schematic overview of the processes of cohesin loading and establishment and homologous chromosome pairing and recombination, is shown in Figure 1.

### Release of chromosome cohesion: separase

Cleavage of the α-klesin subunit occurs in a stepwise manner during meiosis. In meiosis I, Rec8 is cleaved at chromosome arms, allowing the resolution of chiasmata and homologous chromosome segregation in meiosis I, whereas in meiosis II cohesin is released at centromeres, enabling sister chromatid separation (Nasmyth, 2001). Cleavage of Rec8 is performed by the cysteine protease Separase, which is conserved in various organisms, including yeast and vertebrates (Kitajima et al., 2003; Kudo et al., 2009). Separase function is inhibited by a protein called Securin, which is degraded at the onset of anaphase by ubiquitylation by the APC/C (Uhlmann, 2001). Homologs of separase are present in many plant species. However, the studies undertaken so far have only focused on the *Arabidopsis* separase AESP (Liu and Makaroff, 2006). AESP is an essential gene but RNA interference of AESP under the control of the meiotic DMC1 promoter, and the finding of the temperature permissive mutant *rsw4* (radially swollen 4), have allowed to investigate AESP function in meiosis (Liu and Makaroff, 2006; Wu et al., 2010; Yang et al., 2011). *Aesp* and *rsw4* mutants display defective chromosome segregation in meiosis I, in which entangled chromosomes and chromosome fragments are observed, and in meiosis II, where bivalents are still present, indicating persistence of cohesion (Liu and Makaroff, 2006; Yang et al., 2011). In support of this, SYN1 and SMC3 signals persist on *aesp* and *rsw4* chromosomes at later stages after metaphase I, demonstrating that AESP is responsible for removal of the cohesin complex from chromosomes. The creation of a double mutant between *aesp* and *ask1*, in which homologous chromosomes prematurely separate in meiosis I due to defects in homologous synapsis, showed that sister chromatids did not separate in meiosis II. This observation confirms that AESP is responsible for sister chromatid separation also in anaphase II (Yang et al., 2009). In *Arabidopsis*, a large amount of cohesin is released from chromosome arms in prophase I and the residual arm cohesin is released at anaphase I (Cai et al., 2003). While AESP is required for the release of cohesin at anaphase I and in meiosis II, it does not participate in the first step of release in prophase I, suggesting that a separase-independent mechanism might exist at early stages in *Arabidopsis*, similar to budding yeast, in which the condensin complex SMC2/SMC4 and a Polo kinase are responsible for cohesin removal at chromosome arms before metaphase I (Sumara et al., 2002; Yu and Koshland, 2005; Liu and Makaroff, 2006).

Separase is a multifunctional protein that in various organisms possesses additional roles to sister chromatid separation mechanistically less understood, such as proteolytic cleavage of other target proteins in yeast and spindle assembly in humans (Moschou and Bozhkov, 2012). Also in *Arabidopsis* additional functions of separase have been reported (Yang et al., 2009, 2011). *Aesp* mutants show alterations in non-homologous centromere associations at zygotene, suggesting that AESP might play a role in the control/release of the transient centromere associations that occur during zygotene in *Arabidopsis* (Armstrong et al., 2001). Furthermore, in *aesp* male meiocytes the radial microtubule array (RMA) is disturbed at telophase II and phragmoplast-like structures are observed, suggesting that AESP might have a function in microtubule organization or cell polarity (Yang et al., 2009). Absence of AESP also causes the formation of multinucleate microspores as a consequence of defective RMA (Yang et al., 2009). In contrast to yeast, where separase is required for normal meiotic spindle formation (Jensen et al., 2001; Baskerville et al., 2008), in *Arabidopsis* only RMA formation is defective while AESP might be required for the proper interaction of microtubules with the nuclear envelope at the tetrad stage (Yang et al., 2009).

### Protection of centromere cohesion: shugoshin and patronus

In meiosis, sister chromatid cohesion is controlled in a time- and space-dependent manner, with chromosome arm cohesion release at the start of anaphase I, and maintenance of centromeric cohesion up till anaphase II. Meiosis-specific protection of Rec8 at pericentromeric regions from anaphase I to anaphase II is performed by Shugoshin (Sgo), a protein first described in *Drosophila* (MEI-S332; Kerrebrock et al., 1995), and successively identified in yeast, mammals and plants (Yao and Dai, 2012). Studies from yeast and vertebrates have elucidated the mechanism of action of Sgo, which is recruited at pericentromeric heterochromatin regions where it associates with the phosphatase PP2A to dephosphorylate Rec8 and prevent its cleavage in meiosis I (Lee et al., 2008a; Xu et al., 2009). In yeast, Sgo1 localizes at centromeres until the end of anaphase I (Kitajima et al., 2004), whereas in vertebrates SGOL2 persists on the chromosomes also in meiosis II (Lee et al., 2008a). Currently, two hypotheses are postulated to explain the dynamic association of Shugoshin with centromeres. On the one hand, Sgo function may be controlled by microtubule attachment and deactivated by a spatial change of its localization in the peri-centromeric domain in response to a change in microtubule tension (Lee et al., 2008a). Alternatively, a PP2A inhibitor may block dephosphorylation thereby conferring loss of protection of centromeric cohesion in meiosis II (Chambon et al., 2013). Flies and budding yeast possess a single copy of Sgo, while fission yeast, mammal and plant genomes have two Sgo paralogs, Sgo1 and Sgo2. In *Drosophila*, yeasts and plants, Sgo1 is responsible for the protection of centromere-specific sister chromatid cohesion in meiosis I, while in mammals SGOL2 performs the function of protector (Gutiérrez-Caballero et al., 2012). Though they are homologs, Sgo genes share limited sequence similarity and display in the different organisms somewhat different functions which have been acquired during evolution (for a recent review on the Shugoshin protein family and the additional roles of Shugoshin see Clift and Marston, 2011; Gutiérrez-Caballero et al., 2012). The *Sgo1* paralog *Sgo2* possesses different properties depending on the species examined. In fission yeast, Sgo2 plays a role in chromosome segregation in mitosis (Kitajima et al., 2004), in particular it has been shown to control the localization of the CPC, a protein complex that senses lack of tension between kinetochores and microtubules (Kawashima et al., 2007; Vanoosthuyse et al., 2007; Tsukahara et al., 2010). In addition, fission yeast Sgo2 also plays a role in meiosis, as *Sgo2* deletion leads to a modest increase in non-disjunction of homologs at meiosis I (Kitajima et al., 2004). In humans, hSGOL1 protects centromeric cohesion in mitosis (Salic et al., 2004; McGuinness et al., 2005), whereas hSGOL2 is dispensable for sister chromatid cohesion in mitotic cell division but is essential for correcting erroneous kinetochore attachments by recruiting the microtubule depolymerase MCAK to the centromeres (Huang et al., 2007), a role that is consistent with the one shown for fission yeast Sgo2 (Kawashima et al., 2007).

In plants, the role of Sgo as protector of centromere cohesion in meiosis has been described for the maize ZmSGO1, the rice OsSGO1 as well AtSGO1 and AtSGO2 of *Arabidopsis* (Hamant et al., 2005; Wang et al., 2011; Cromer et al., 2013; Zamariola et al., 2013, 2014). FISH analysis performed on *sgo1* meiocytes with a centromeric probe revealed a premature detachment of sister chromatid centromeres in anaphase I, resulting in random chromosome segregation in meiosis II. However, monopolar orientation of sister kinetochores in meiosis I is not affected in the mutants and chromosomes normally segregate in the reductional division, indicating that SGO proteins are required for protection of cohesion at anaphase I but not for monopolar orientation of sister kinetochores. In fission yeast and mammals, which possess two Sgo homologs, one copy is generally required for protection of sister chromatid cohesion in meiosis, while the other has evolved additional roles, as previously mentioned. So far, no function in somatic cells has been described for any of the plant Sgo proteins. *Arabidopsis* is the only species in which the role of both Sgo paralogs has been investigated. Single mutants show no vegetative phenotype and a meiotic phenotype is detected exclusively for *Atsgo1*. However, *Atsgo1 Atsgo2* double mutants reveal a partially redundant role for the two SGOs, opposite to yeast and vertebrate (Cromer et al., 2013; Zamariola et al., 2014). Immunolocalization of ZmSGO1 and OsSGO1 has revealed that SGO1 is loaded on chromosomes at leptotene, earlier than in other organisms such as yeast or mammals in which loading occurs during late prophase I or at diplotene, respectively (Kitajima et al., 2004; Gómez et al., 2007). Thus, plant SGO proteins might have a function in prophase I. In support of this hypothesis, ZEP1 localization is defective in *Ossgo1* mutants in about 21% of meiocytes, indicating that OsSGO1 may be required for the timely assembly of the SC, even if not for its initial assembly (Wang et al., 2011). In contrast, *Arabidopsis* ZYP1 localizes normally in *Atsgo1* mutants (Zamariola et al., 2013).

Recently, a novel protein involved in the protection of sister chromatid cohesion during meiosis II has been identified in *Arabidopsis*, named PATRONUS (PANS1) (Cromer et al., 2013; Zamariola et al., 2014). PANS1 is a plant specific protein that shares homology with genes belonging to the Eudicots family. *Pans1* meiocytes show a premature release of sister chromatid cohesion at metaphase II but not at meiosis I, indicating that the protein is required for protection of cohesion during interkinesis, at a later stage than SGOs. Moreover, similar to SGOs, PANS1 is not required for monopolar attachment of sister kinetochores in meiosis I. TAP-TAG and Y2H experiments have revealed that PANS1 may be a regulator of the APC/C complex because of the interaction with some of the APC/C subunits. In addition, the presence of two destruction boxes in the PANS1 sequence may indicate that PANS1 is at the same time also targeted by the APC/C complex. Currently, three hypotheses have been suggested to explain how PANS1 maintains sister chromatid cohesion at interkinesis: (1) by protecting SGOs from destruction by the APC/C; (2) by protecting sister chromatid cohesion from Separase independently of SGOs, in the case SGOs are no longer present after anaphase I, and (3) by inhibiting via APC/C regulation the Wapl-dependent process of cohesin release, which is usually activated at the end of mitosis/G1 phase to allow dynamic cohesin renewal and that could be present also at the end of meiotic telophase I (Cromer et al., 2013). At the moment, AtSGOs and PANS1 localization, that could help unraveling the function of PANS1 in meiosis and the relation among the protectors, is lacking. Besides its role as protector of cohesion, PANS1 has also been shown to be required for spindle organization in meiosis since *pans1* meiocytes display defective spindles starting from telophase I. Defective spindles is probably the cause of the formation of an aberrant internuclear organelle band at interkinesis, detected in 7% of *pans1* meiocytes. Taken together, these phenotypes and the premature separation of sister chromatids observed in meiosis II, suggest a function of PANS1 in ensuring the coordinate organization of the cell organelles in accordance with the meiotic cell cycle phase and chromosome cohesion (Zamariola et al., 2014), which is in agreement with the interaction of PANS1 with the APC/C.

## Centromeres and kinetochores

### Role of centromeres and kinetochores in chromosome segregation

Centromeres are DNA-protein structures necessary to direct chromosome movement in cell division. Centromere DNA sequences are fast evolving and highly variable among species. However, centromeric regions in most plant species encompass mainly two domains. One is the core centromere, which contains satellite tandem repeats, usually 150–180 bp long, and specialized nucleosomes in which histone H3 is replaced by a centromere-specific H3 histone variant, CENH3. This region is required for the assembly of the kinetochore, a protein structure that binds to spindle microtubules allowing faithful chromosome segregation. The core centromere is flanked by pericentromeric heterochromatin domains containing retroelements and other transposons. In yeast the pericentromeric domains have been shown to have mainly a role in the recruitment of Shugoshin (Pidoux and Allshire, 2005; Yamagishi et al., 2008). In addition, epigenetic mechanisms may be involved in the specification of centromeric chromatin and propagation of centromeres (Houben and Schubert, 2003; Ekwall, 2007; Torras-Llort et al., 2009; Wang et al., 2009).

The specific centromeric variant Histone 3, CENH3, was first identified in human as CENP-A and subsequently in all eukaryotic model systems (De Rop et al., 2012), including *Arabidopsis* (also called HTR12; Talbert, 2002). Despite its essential and conserved role in ensuring proper chromosome segregation, CENH3 proteins are highly variable in their sequences and fast evolving, especially their N-terminal tail domain and a loop 1 region at the C-terminal domain, which are necessary for CENH3 localization to centromeres in *Arabidopsis* (Ravi et al., 2010; Moraes et al., 2011). The C-terminal part of the protein is sufficient for the centromeric localization of CENH3 in mitotic cells even when the N-terminal part is absent (Lermontova et al., 2006). In meiosis, a different loading mechanism for CENH3 is present, in which the N-terminal tail plays a critical role. *Arabidopsis* plants transformed with a N-terminally truncated YFP-CENH3(C) protein show meiotic defects and partial sterility and the YFP signal cannot be detected in meiotic nuclei (Lermontova et al., 2011). Similarly, the replacement of the N-terminal tail with a GFP tagged variant, GFP-tailswap, causes sterility due to defects during sporogenesis (Ravi et al., 2011). In GFP-tailswap plants, meiosis is disturbed starting from metaphase I, in which bivalents align on the division plate but are not subjected to tension from the spindle, which is confirmed by decreased interkinetochore distance and by defective spindles (Ravi et al., 2011). CENH3 protein signal is reduced or not detected in GFP-tailswap meiocytes and is again detected after meiosis on mitotic chromosomes at the microspore stage, indicating the existence of distinct mechanisms for CENH3 loading in meiosis and mitosis (Ravi et al., 2011). The work of Lermontova et al. (2011) also suggests a different loading mechanism in meiosis and mitosis, since the YFP-CENH3(C) variant is deposited to the centromeres in mitosis but not in meiotic nuclei.

Recently, the *Arabidopsis* homolog of KNL2 has been identified. It represents one of the components of the Mis18 complex, responsible for the initiation of CENH3 deposition at the centromeres in humans (Hayashi et al., 2004), *C. elegans* (De Rop et al., 2012) and fission yeast (Hayashi et al., 2004). In *Arabidopsis*, KNL2 is associated with centromeres at all stages of the cell cycle except from metaphase to mid-anaphase. *Arabidopsis* KNL2 knockout mutants show defects in mitosis and meiosis and reduced CENH3 loading at the centromeres (Lermontova et al., 2013). Furthermore, CENH3 gene expression is decreased in *knl2* mutants but KNL2 expression is stable in CENH3 RNAi transformants, indicating that KNL2 acts upstream of CENH3 and has a function in the assembly of CENH3 at the centromeres (Lermontova et al., 2013). Moreover, KNL2 is co-expressed with H3K9 histone methyltransferases genes, whose expression is reduced in *knl2* mutants. Also DNA methylation levels are lower in *knl2* mutant plants. The requirement of KNL2 for CENH3 expression and for DNA methylation, suggests that KNL2 may interact with methyltransferases to allow the maintenance of DNA methylation, in order to control the epigenetic status of centromeric chromatin and to control CENH3 loading (Lermontova et al., 2013).

Sister kinetochores must behave differently in meiosis I and II: in meiosis I are oriented toward the same pole (mono-orientation) to allow homologous chromosomes segregation, while in meiosis II they face opposite poles (bi-orientation) (Brar and Amon, 2009). The tension exerted at the kinetochores by microtubules during division, and the kinetochore geometry, defined in meiosis and mitosis by cohesion, are fundamental for stabilizing the monopolar attachment in MI and the bipolar in MII (for review see Watanabe, 2012). In contrast to the high variability of centromeric sequences, more than 20 active kinetochore proteins are conserved between humans and yeasts (Lampert and Westermann, 2011), and are specific either for the inner kinetochore, where they directly recognize and bind DNA, or for the outer kinetochore, being responsible for the interaction with microtubules (Santaguida and Musacchio, 2009; Wang et al., 2009). However, to date, only 7 kinetochore proteins have been reported to be conserved in *A. thaliana*, the majority of which has not been yet functionally characterized (Murata, 2013). The inability to identify homologs of many human and yeast kinetochore proteins in plants, may suggest the existence of different kinetochore structure in plants (Murata, 2013).

Kinetochore functionality depends on the presence of a functional centromere in meiosis. Indeed MIS-12, a kinetochore protein which co-localizes with CENH3 at the centromere regions (Sato et al., 2005), does not do it in *Arabidopsis* GFP-tailswap meiocytes. In contrast, CENP-C, another kinetochore protein which localizes at the centromeres in mitotic cells (Ogura et al., 2004), is not affected in CENH3 RNAi transformants, suggesting that its localization does not depend on the presence of a functional CENH3 (Lermontova et al., 2011).

**Figure 2:**
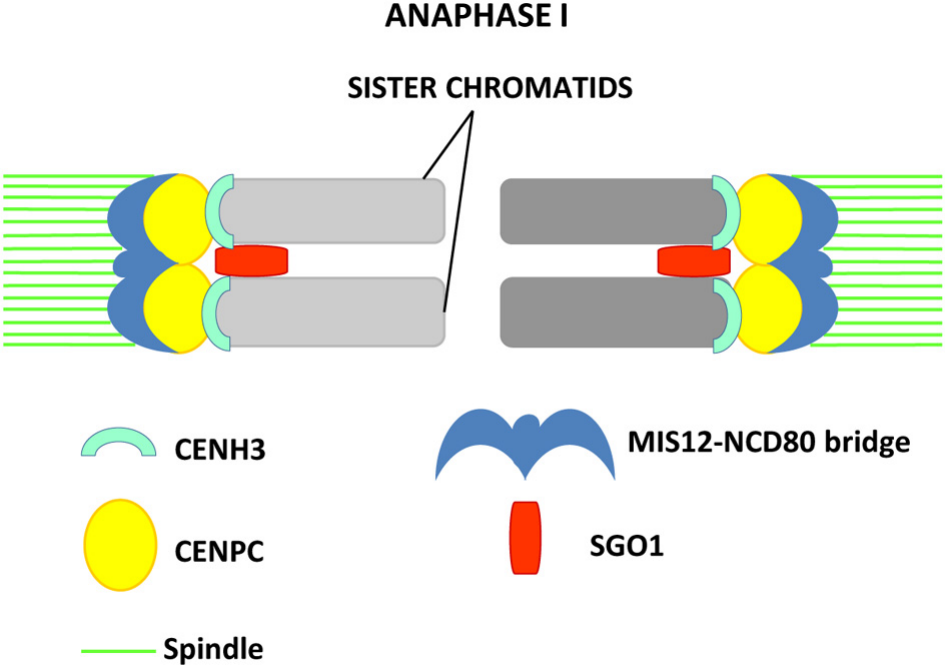
**Model proposed in maize by Li and Dawe (2009) for reductional segregation in meiosis I**. Sister kinetochores are fused in meiosis I by formation of the MIS12-NCD80 bridge that, together with SGO1, allows monopolar attachment of sister chromatids to the spindle pole. The inner kinetochore proteins CENPC and CENH3 are visualized as two distinct signals.

In maize, kinetochore proteins have been more thoroughly investigated. CENPC is part of the inner kinetochore and interacts at one side with the DNA repeats located at the centromeric regions, and, on the other side, with the members of the outer kinetochore (Dawe et al., 1999; Zhong et al., 2002). At the outer kinetochore NCD80 and MIS12 are present. Homologs of these two proteins are known to be parts of the KMN (KNL-1/Mis12/Ndc80) complex that constitutes the core microtubule-binding site of the kinetochore in *C. elegans* (Cheeseman et al., 2006). NDC80 is a constitutive kinetochore protein which localizes at kinetochores in all meiotic and mitotic stages (Du and Dawe, 2007). It does not bind DNA directly and interacts with MIS12, which is also present at kinetochores during all stages of the cell cycle. NCD80 and MIS12 form at metaphase I a bridge structure that links sister kinetochores, while CENH3 and CENPC appear at the inner side of sister kinetochores as two distinct signals (Li and Dawe, 2009). MIS12 has an important role in sister chromatid connection at meiosis I and is required for the initiation of reductional division (Li and Dawe, 2009). Knock-down of MIS12 by RNAi leads to a weakening of the MIS12-NCD80 bridge and aberrant chromosome segregation in meiosis I, where in 30% of the cells sister kinetochores separate and segregate in an equational division instead of reductional (Li and Dawe, 2009). In MIS12 RNAi cells, the signal of the centromere protector ZmSGO1 does not weaken (Li and Dawe, 2009). The protein lies between sister kinetochores but cannot restore kinetochore co-orientation, confirming that Shugoshin is not required for the monopolar orientation of kinetochores (Hamant et al., 2005; Li and Dawe, 2009). A model, in which axial elements and cohesin hold sister chromatids together during prophase I and create the base for fused sister kinetochore formation promoted by the MIS12-NCD80 bridge has been proposed (Li and Dawe, 2009). This structure would cooperate with Shugoshin to induce reductional segregation by co-orienting sister kinetochores (Figure 2). MIS12 and NCD80 are thought to be similar to the monopolin complex, which promotes sister kinetochore co-orientation in budding yeast (Corbett and Harrison, 2012).

### Checkpoint mechanisms acting at kinetochores

In eukaryotes, checkpoint mechanisms are present in meiosis and mitosis to prevent chromosome mis-segregation that would result in aneuploidy or apoptosis (Murray, 1994). The SAC is a conserved protein complex that controls proper attachment of microtubules to kinetochores in the metaphase to anaphase transition. In case of lacking or improper kinetochore-microtubule attachment, SAC creates a “wait anaphase” signal that stops anaphase progression. This response is promoted by APC/C together with its co-activator Cdc20 protein (for reviews see Peters, 2006; Vader et al., 2008; Musacchio, 2011). When all kinetochores are properly attached to microtubules, APC/C targets the destruction of Securin, the inhibitor of Separase as well as other cyclins, promoting chromosome segregation and exit from meiosis or mitosis. Evolutionary conserved proteins of SAC are MAD1, MAD2 (mitotic arrest deficient), Bub1, Bub3 (budding unhibited by benomyl), BubR1 kinase (bub-related1, Mad3 in yeast), and Mps1 (Monopolar kinase1) (May and Hardwick, 2006). The SAC proteins BubR1, Bub3, and MAD2 are also members of the Mitotic Checkpoint Complex (MCC), which is the effector of SAC that physically inhibits APC/C by binding to its co-activator Cdc20 until the moment all chromosomes are properly attached to kinetochores (Sudakin et al., 2001). SAC function has been investigated in depth in mitosis. However, a similar control mechanism is active also during meiosis (Malmanche et al., 2006; Sun and Kim, 2012).

Homologs of SAC proteins have been described in plants. MAD2 was first identified in maize where it localizes to the outer kinetochore in prometaphase I and II of meiosis, next to the inner kinetochore protein CENPC (Yu et al., 1999). During meiosis I and II, microtubule attachment is not sufficient for MAD2 dissociation from kinetochores, and the dissociation might occur in response to tension applied to the kinetochores (Yu et al., 1999). This hypothesis is supported by the concomitant staining of the MAD2 and 3F3/2 antibodies in maize meiosis. 3F3/2 recognizes a kinetochore phosphoepitope that is known to disappear in animal cells when tension is applied to the kinetochore (Nicklas et al., 1995). MAD2 homologs have been identified also in wheat and *Arabidopsis*, and their roles have been mainly investigated in mitotic checkpoint control. In wheat, intense MAD2 signal was observed at all centromeres in colchicine treated cells but not in untreated cells, confirming the function of MAD2 in the spindle checkpoint (Kimbara et al., 2004). In *Arabidopsis*, MAD2 localization was studied together with BubR1 and Bub3.1, the others SAC proteins identified in the model plant (Caillaud et al., 2009). Interactions between the three proteins were observed in the nuclei of tobacco cells using bimolecular fluorescence complementation (Caillaud et al., 2009). During normal mitosis, localization of the SAC proteins to the kinetochores was not detected. However, by application of microtubule destabilizing drugs or of the proteasome inhibitor MG132, MAD2, BubR1, and Bub3.1 localized at the kinetochores, suggesting that SAC proteins are only recruited at kinetochores in case of defective spindle assembly in *Arabidopsis* (Caillaud et al., 2009). In contrast, a study by Ding et al. (2012) on the *Arabidopsis* MAD2 protein, showed that MAD2-GFP localizes at kinetochores also during normal mitotic progression from prophase to metaphase, as shown in maize. Moreover, AtMAD2 binds to AtMAD1, which interacts with the nucleoporin NUA, showing that SAC components interact with the nuclear pore. This interaction has been found in several other organisms, and it seems that the presence of SAC proteins at the nuclear pore mediates mitotic spindle checkpoint (Lee et al., 2008b).

Mps1 (Monopolar kinase 1) is also required for SAC function in the mitotic checkpoint in several eukaryotes and has been shown to be responsible for the recruitment of Mad1 and Mad2 at kinetochores in humans (Hewitt et al., 2010). Mps1 *Arabidopsis* homolog has conserved motifs which could mediate its interaction with MAD2 but also with cyclins, the APC/C and MAPK (mitogen-activated protein kinases), however, proof of its biological role in the checkpoint mechanism is still required (De Oliveira et al., 2012).

In most organisms, SAC is controlled by the chromosome passenger complex (CPC). In general, the CPC consists of the core enzyme Aurora B kinase, and three non-enzymatic subunits that control the targeting, enzymatic activity and stability of Aurora B: inner centromeric protein (INCENP), borealin and survivin (for review see Ruchaud et al., 2007). The major role of CPC is sensing incorrect kinetochore-microtubule attachments and generating, in response, unattached kinetochores, which allows new rounds of attachment until the correct configuration is obtained. The presence of unattached kinetochores activates the SAC that blocks the progression of cell divisions until all chromosomes are under tension. In plants, little is known about the role of the CPC in meiosis and few components of the complex have been identified. Like animals, *Arabidopsis* possesses three Aurora kinase homologs, which share a similar structure to the ones of other species (Kawabe et al., 2005). AtAurora1 and AtAurora2 display similar localization dynamics to Aurora B kinase in *Arabidopsis* mitosis, suggesting that they could function as chromosomal passenger proteins (Demidov et al., 2005). AtAurora1 interacts with SAC proteins BubR1 and MAD2 *in vivo* and phosphorylate them *in vitro*, which suggests that it functions in checkpoint mechanisms (Demidov D., personal communication). Furthermore, deregulation of AtAurora kinases activity, either by mutagenesis or by chemical treatment, results in defects in microsporogenesis and generation of polyploid and aneuploid progeny, suggesting that AtAurora may regulate correct chromosome segregation in *Arabidopsis* meiosis (Demidov D., personal communication).

A putative ortholog of the CPC subunit INCENP, WYR, has been identified in *Arabidopsis* (Kirioukhova et al., 2011). WYR shares with the INCENP homolog proteins a characteristic C-terminal domain, a coiled coil domain and a IN-box at the C-terminus, required for the binding of Aurora kinase. WYR is an essential gene with a role in cell cycle control and, independently, in cell fate and differentiation in *Arabidopsis*, since is required for both female and male gametogenesis. Similar functions have been reported also for the orthologs of INCENP in animals (Ruchaud et al., 2007). However, further genetic and biochemical analyses on WYR and Aurora kinases are required to establish the role of CPC proteins in plants.

## Microtubule organization and spindle dynamics

In all eukaryotic cells, faithful chromosome segregation is accomplished by microtubule-based movement and requires a bipolar structure, the spindle, which consists of an antiparallel array of microtubules. The microtubules have their minus-end anchored at the spindle pole and their plus-end toward the chromosomes (Wittmann et al., 2001). They are highly dynamic polar polymers of noncovalently bound α and β tubulin heterodimers and represent the major components of the cytoskeleton in eukaryotic cells (Nogales, 2000). They rapidly polymerize and depolymerize while being continually translocated toward the poles. In animal and yeast cells, microtubules nucleate from microtubule-organizing centers (MTOC), such as the centrosome and the spindle pole body, which are responsible for the organization of the cortical astral arrays in interphase and mitotic spindles during cell division (Pereira and Schiebel, 1997; Jaspersen and Winey, 2004). γ-tubulin is enriched at the nucleation centers where it is recruited as a ring-shaped complex together with associated proteins, enhancing the nucleation of microtubules (O'Toole et al., 2012). In contrast to animals and yeast, plant microtubules lack conspicuous organizing centers. However, they are organized into ordered arrays that are associated with a growth pattern of the plant cell and relocate in a cell-cycle specific manner (Azimzadeh et al., 2001). During cell division, a succession of microtubule arrays is identified: radial arrays from the nuclear surface and cortical arrays of interphase, preprophase bands, spindles, and phragmoplasts (Wasteneys, 2002; De Storme and Geelen, 2013a). Like in animal and yeast, γ-tubulin is also required for microtubule nucleation in plants, being essential for the organization of the microtubule structures in interphase and cell division (Canaday et al., 2000; Shimamura et al., 2004; Pastuglia et al., 2006).

Microtubule motor proteins have an essential role in spindle assembly in both centrosomal and acentrosomal systems (Walczak et al., 1998). The best studied class of microtubule motor proteins are kinesins, proteins that participate in a variety of biological processes, including transport of vesicles, chromosomes or organelles, and organization of spindle microtubules, and chromosome segregation (Woehlke and Schliwa, 2000). They move unidirectionally along microtubules toward their plus or minus-ends. They use energy derived from ATP hydrolysis, in a processive or non-processive way, depending on their capacity of moving cargo long or only short distances before detaching from the microtubules. Several kinesins are known to be required for the structure, assembly and positioning of the mitotic and meiotic spindles in animals and fungi (Endow, 1999; Sharp et al., 2000). The *Arabidopsis* genome contains 61 predicted kinesins, one-third of them belonging to the kinesin-14 family that includes minus end-directed motor proteins (Reddy and Day, 2001). ATK1 is a member of this family and has been shown to support microtubule movement in an ATP-dependent manner and to be a non-processive, minus-end motor protein (Marcus et al., 2002). ATK1 has a specific role in male meiosis, in which *atk1-1* meiocytes display defective chromosome alignment and segregation in meiosis I and II due to aberrant formation of metaphase and anaphase spindles, leading to spore and pollen abortion and decreased plant fertility (Chen et al., 2002). ATK1 is involved in the assembly of the meiotic spindle and is needed for organizing microtubules at the two poles at metaphase and anaphase I and II, but not for the organization of microtubules for other structures, such as the interzonal microtubule array formed at telophase I (Chen et al., 2002). Studies in yeast and *Drosophila* have suggested that minus and plus-ended motor proteins could produce counteracting forces within the spindle to maintain its structure (Sharp et al., 1999, 2000). Thus, ATK1 might have a similar function in plant male meiosis, by producing inward-acting forces necessary for the assembly and maintenance of a bipolar spindle (Chen et al., 2002). The creation of a double heterozygote mutant between ATK1 and its homolog ATK5 (also named AtKIN14a and AtKIN14b, respectively), has shown that both proteins are required for proper chromosome segregation in female and male meiosis and for normal spindle morphogenesis in male meiosis (Quan et al., 2008). In addition to its male meiotic function, ATK1 localizes to the midzone of the mitotic spindle from metaphase through anaphase, suggesting a function also in the mitotic spindle apparatus (Liu et al., 1996).

AtPRD2/MPS1 (Multi-polar spindle1) is a putative *Arabidopsis* coiled-coil protein with homologs only among Embryophytes. Although having been identified as AtPRD2, an essential protein for DSBs formation, due to the presence of univalents in *Atprd2* mutant meiosis (De Muyt et al., 2009), the protein has also been found to be required for spindle organization and determination of spindle polarity in male meiosis (MPS1; Jiang et al., 2009). *Mps1* meiocytes display multiple focused spindles at metaphase I, indicating that spindle assembly is not defective, in contrast to *atk1* and *atk1/atk5* mutants, but spindle bipolarity is compromised in meiosis I and II, and chromosome segregation results more affected than in the kinesin mutants. This observations suggest that MPS1, ATK1, and ATK5 play a role in different mechanisms in plant meiosis. It has been proposed that MPS1 might guide microtubule minus-end migration in meiosis, maybe through binding to an unknown MAP (microtubule associated proteins) or, alternatively, could be a component of the spindle pole transmitting the signal to attract the minus-end of the spindle microtubules before spindle assembly (Jiang et al., 2009). However, whether the spindle defects observed in *mps1* meiocytes correspond to a primary function of the protein in spindle organization and polarity, or to a secondary effect caused by univalents formation in meiosis I, is not clear since conflicting observations on the relationship between unpaired chromosomes and spindle aberrations have been reported (Chan and Cande, 1998; Dawe, 1998).

In rice, a Kinesin-1-like protein, Pollen Semisterility 1 (PSS1), has been shown to have microtubule-stimulated ATPase activity and to be required for proper chromosome alignment and segregation in meiosis. However, spindle morphology is only slightly affected in *pss1* mutants, indicating that PSS1 might have a minor and not essential role in the formation of the meiotic spindle or alternatively might be involved in the regulation of chromosome movements along the spindles, as suggested by the delayed chromosomes observed in meiosis in *pss1* (Zhou et al., 2011).

Recently, the identification of a MATH-BTB domain protein, MAB1 (MATH-BTB1) in maize has been reported. This protein is required for organizing microtubule spindles and nuclei positioning in meiosis II and in the first mitotic division in both male and female germlines. Since no direct interaction between MAB1 and the spindles has been observed, it has been proposed that MAB1 may act through the control of a spindle apparatus regulator(s) (Juranič et al., 2012). Six MATH-BTB proteins have been currently identified in the *Arabidopsis* genome, however, no similar function has been reported (Weber and Hellmann, 2009).

The correct orientation of spindles in the second meiotic division is an essential requirement for faithful chromosome segregation. Alterations in the orthogonal configuration of the division planes in meiosis II lead to co-orientation of the spindles producing unreduced gametes, that represent the major route to polyploidization in plants (Brownfield and Köhler, 2011). Co-orientation can lead to the formation of three types of MII spindle defects which usually occur together in cells: parallel, tripolar or fused (Conicella et al., 2003; De Storme and Geelen, 2013b). This phenomenon only takes place in PMCs (pollen mother cells) of plants with simultaneous cytokinesis. In this type of cytokinesis, as opposed to the successive type, no cell plate is formed at the end of meiosis I and the two sets of chromosomes stay in the same cytoplasm and need to be perpendicularly oriented to create the tetrahedral configuration observed at the end of meiosis II (De Storme and Geelen, 2013a). They have been documented in many plant species, however, the molecular mechanisms behind their occurrence are still largely unknown. Two proteins involved in spindle orientation specifically in male meiosis II have been identified in *Arabidopsis*: AtPS1 and JASON (D'Erfurth et al., 2008; Erilova et al., 2009; De Storme and Geelen, 2011). Mutations in these genes produce at the end of meiosis II a high number of unreduced gametes (i.e., dyads and triads) instead of normal haploid gametes, leading to diploid pollen formation and triploid offspring. The biological mechanism causing 2n gamete formation in the mutants has been elucidated by tubulin immunostainings, which have shown the formation of parallel, tripolar and fused spindles in meiosis II. The defective spindles lead to 2n spores that retain parental heterozygosity at the centromeres, indicative of a FDR-type (first division restitution) of meiotic restitution (D'Erfurth et al., 2008; De Storme and Geelen, 2011). The introduction of *Atsp1* or *jason* mutations into the *Atspo11* mutant background has confirmed the model of 2n gametes formation through co-oriented spindles, since the unbalanced segregation caused by *Atspo11* at meiosis I is nullified by parallel spindles in meiosis II, leading to the formation of mainly balanced dyads as result of meiosis in the double mutants. *Atps1* and *jason* meiocytes lack the characteristic interzonal microtubule array (IMA) observed in simultaneous PMCs at telophase I, which physically separates the two new formed nuclei. They mostly show fused nuclei at metaphase II. In potato, the absence of IMA has also been proposed to cause alterations in cell polarity and the formation of fused spindles (Conicella et al., 2003), suggesting that also in the *Arabidopsis* mutants depending on the total, partial, or unipolar loss of IMA fused, parallel or tripolar spindles are formed (De Storme and Geelen, 2013b).

AtPS1 is a protein conserved in the plant kingdom (Cigliano et al., 2011), which contains two conserved domains in its structure: an N-terminal Forkhead-associated (FHA) domain required for phosphoprotein interaction in many signaling pathways (Li et al., 2000) and a PINc domain that has RNA-binding properties associated with RNAse activity, and which is generally found in proteins involved in RNAi and in nonsense-mediated mRNA decay (NMRD) (Clissold and Ponting, 2000). JASON encodes a protein of unknown function and no known domains that is conserved in plants (Erilova et al., 2009). Expression analysis have demonstrated that *JASON* controls the *AtPS1* transcript level specifically in meiotic flower buds, suggesting the existence of a regulatory mini-network for the control of spindle orientation in meiosis II (De Storme and Geelen, 2011).

Defects in spindle orientation in the second meiotic division have been also reported in mutants in one of the *Arabidopsis* formins, AFH14 (Li et al., 2010). Formins are a class of proteins known to regulate the microfilament cytoskeleton (Blanchoin and Staiger, 2010), but have been recently shown to have also a prominent role in microtubule regulation and in the crosstalk between actin filaments and microtubules in higher eukaryotes (Bartolini and Gundersen, 2010). Indeed, microtubules and microfilaments have been shown to co-distribute and interact in the meiotic spindle and in the phragmoplast in maize (Staiger and Cande, 1991). AFH14 co-localizes with MTs and MFs arrays during cell division in *Arabidopsis* suspension cells and with MTs in meiotic cells, affecting their arrangement during microsporogenesis. *Afh14* mutants display abnormal MTs structures including defective RMS at telophase I, parallel spindles at metaphase II and the absence of phragmoplast structures at late cytokinesis. AFH14 has been shown to preferentially bind MTs and to link MTs and MFs *in vitro*, thus playing a key role in cytoskeletal dynamics and organization required for cell division, including MII spindle orientation (Li et al., 2010; De Storme and Geelen, 2013b).

**Figure 3:**
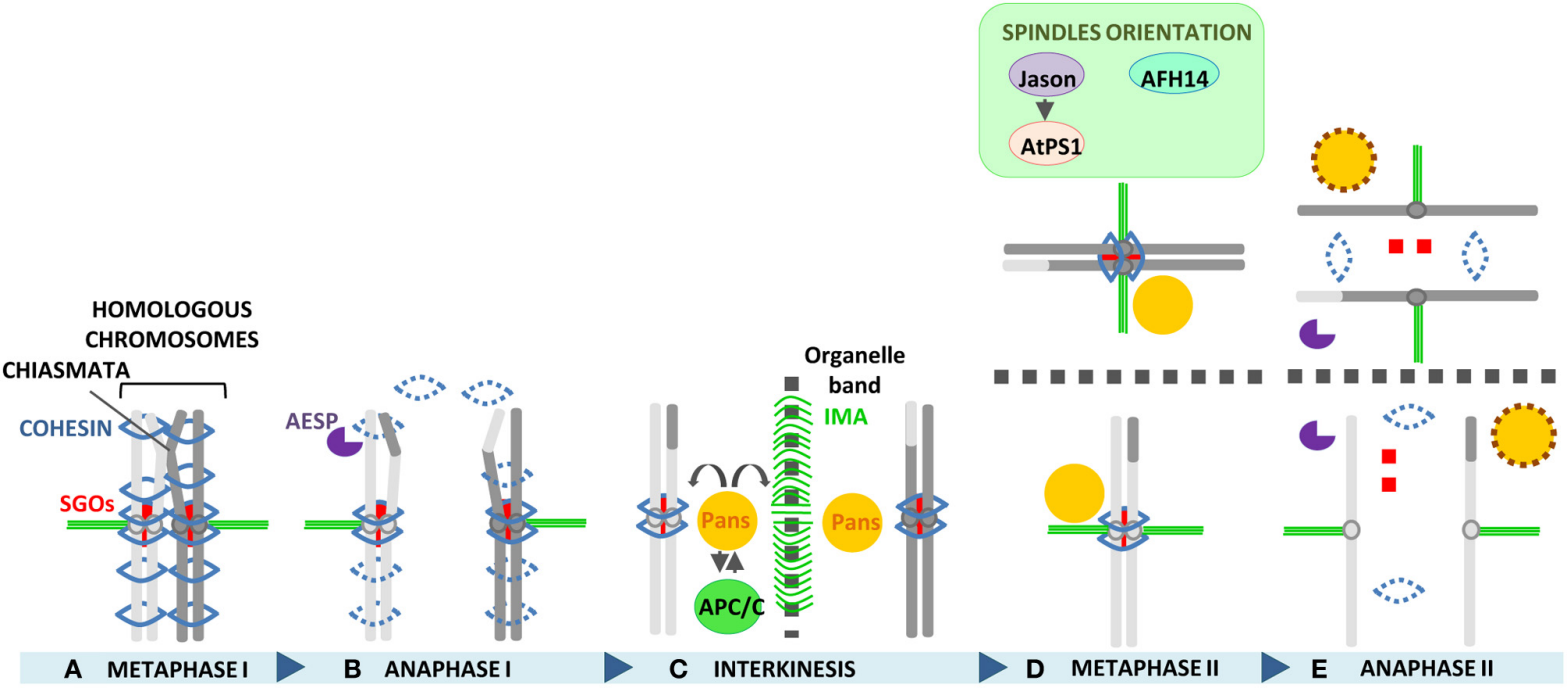
**Overview of chromosome segregation in *Arabidopsis* meiosis. (A)** At metaphase I, homologous chromosomes are connected by chiasmata and SHUGOSHINs (AtSGOs) are present at the centromeres. **(B)** At anaphase I, Separase AESP is activated and cleaves the cohesin at chromosome arms but not at centromeres, allowing resolution of chiasmata and homologous chromosomes segregation by monopolar attachment to the spindles. **(C)** At interkinesis, an internuclear microtubule array (IMA) is formed at the site of the organelle band, to physically separate homologous chromosomes. PANS1 is active and protects centromere cohesin, probably in conjunction with SGOs. PANS1 also interacts with the APC/C, and it is probably also an APC/C target. In addition, PANS1 plays a role in spindle organization from telophase I to telophase II. **(D)** At metaphase II, the chromosomes orient perpendicularly to the metaphase plate through the perpendicular orientation of spindles regulated by Jason and AtPS1. Also the formin AFH14 influences spindle orientation by linking MTs and MFs. **(E)** Releasing or degradation of SGOs and PANS allows cleavage of centromeric cohesin by separase and sister chromatids segregation.

An overview of the process of chromosome segregation between metaphase I and anaphase II, and of the molecular factors playing an essential role in *Arabidopsis* chromosome segregation, is displayed in Figure 3.

## Conclusions and perspectives

In the past 15 years the identification and characterization of plant meiotic genes has seen a remarkable acceleration due to the forward and reverse genetics strategies used in the model plants *Arabidopsis*, maize and rice. In addition, investigation of the molecular mechanisms regulating meiosis in other kingdoms has enormously contributed to the development of plant research in this field. The coordinate events leading to accurate chromosome segregation have been elucidated in budding yeast and studies in plants have confirmed the conserved role of many proteins in the steps of meiotic chromosome segregation, such as cohesin and the dynamics of cohesin removal and protection, the machinery of homologous pairing and recombination, and the function of kinetochores and microtubules. However, even if the main genes have been identified and their function in chromosome segregation confirmed, not much is known about their regulation in accordance with the cell cycle. Further research should focus on investigating the molecular mechanisms regulating protein functions and the interaction between the proteins to define their role in the broader context of chromosome segregation.

### Conflict of interest statement

The authors declare that the research was conducted in the absence of any commercial or financial relationships that could be construed as a potential conflict of interest.
